# Immunologische Aspekte und Stressregulation bei Fatigue

**DOI:** 10.1007/s00103-024-03952-z

**Published:** 2024-09-26

**Authors:** Eva Milena Johanne Peters

**Affiliations:** Psychoneuroimmunologie Labor, Klinik für Psychosomatik und Psychotherapie, Justus-Liebig-Universität Gießen, Aulweg 123, 35385 Gießen, Deutschland

**Keywords:** Fatigue, Immunantwort, Stressreaktion, Psychoneuroimmunologie, Biopsychosozial, Fatigue, Immune response, Stress reaction, Psychoneuroimmunology, Biopsychosocial

## Abstract

Fatigue ist ein Begriff, der eine körperliche messbare, meist muskuläre oder eine empfundene Erschöpfung beschreibt. Fatigue als Zustand wird bei einer breiten Palette langfristiger Belastungen beobachtet, wie etwa bei chronisch infektiösen, autoimmunen oder Krebserkrankungen, sowie bei psychischen Störungen. In diesem Artikel wird ein Überblick zu den bislang bekannten biopsychosozialen Zusammenhängen zwischen Fatigue, psychosozialer Belastung, Stress- und Immunantwort gegeben. Es wird herausgearbeitet, wie chronische Entzündungsprozesse und Stress bei Fatigue interagieren und für welche therapeutischen Ansätze bislang Evidenz vorliegt.

Gemäß dem aktuellen psychoneuroimmunologischen Wissensstand und dem biopsychosozialen Modell können sowohl hohe körperliche und als auch psychosoziale Belastungen in einer neuroendokrin-immunologischen Dysregulation konvergieren. Die Fatigue-Symptomatik korrespondiert nach diesem Modell mit einer chronisch überaktivierten angeborenen Immunantwort. Bei chronischer Immunaktivierung wird zudem eine Fehlaktivierung der erlernten Immunantwort begünstigt, die von (Auto‑)Antikörperproduktion und hyperaktivierten T‑Lymphozyten dominiert wird. Patient*innen, die von Fatigue berichten, weisen jedoch nicht notwendigerweise immunologische Dysregulationen auf. Hier besteht aktuell Forschungs- und Aufklärungsbedarf, um Subpopulationen von Patient*innen und spezifisch zugeschnittene Behandlungskonzepte zu identifizieren.

## Abstract (en)

Fatigue is a term that describes exhaustion as either physically measurable, usually muscular, or perceived. Fatigue as a condition is observed in a wide range of long-term stresses such as chronic infectious, autoimmune, or oncologic diseases as well as mental disorders. This article provides an overview of the currently known biopsychosocial interactions between fatigue, psychosocial stress, and immune response. It describes how chronic inflammatory processes and stress interact in fatigue and for which therapeutic approaches there is evidence to date.

Given the current psychoneuroimmunological knowledge and the biopsychosocial model, both high physical and psychosocial stress can converge in neuroendocrine-immunological dysregulation. According to this model, symptoms of fatigue correspond to a chronically overactivated innate immune response. At the same time, chronic immune activation favors a misactivation of the learned immune response, which is dominated by (auto)antibody production and hyperactivated T lymphocytes. However, patients who report fatigue do not necessarily display immunological dysregulations. There is currently a need for research and education in order to identify patient subpopulations and specifically tailored treatment concepts to them.

## Einleitung

**Abb. 1:**
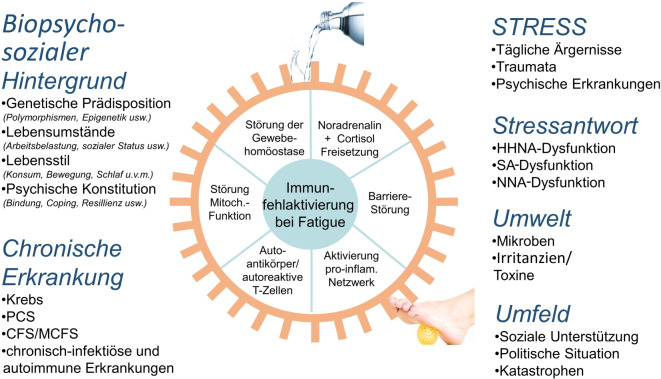
Psychoneuroimmunologische Implikationen von Fatigue. *CFS/MCFS* Chronic Fatigue Syndrom/myalgische Enzephalomyelitis, *HHNA* Hypothalamus-Hypophysen-Nebennierenrinden-Achse, *NNA* Neurotrophin-Neuropeptid-Achse, *PCS* Post-Covid-Syndrom, *SA* sympathische Achse. (Quelle: eigene Abbildung)

Fatigue ist ein Phänomen, dass bei einer ganzen Reihe von chronischen, die Gesundheit beeinträchtigenden Zuständen beschrieben wird [1]. Zu diesen Zuständen gehören chronische Entzündungen, sowohl infektiöse als auch autoimmune, Krebserkrankungen sowie psychische Erkrankungen wie die Depression. Dieses breite Spektrum an assoziierten Erkrankungen gibt bereits einen deutlichen Hinweis darauf, dass keine einheitliche Pathogenese zu erwarten ist. Es weist jedoch auch auf einen möglichen gemeinsamen pathogenetischen Nenner hin: Patient*innen mit diesen Erkrankungen zeigen häufig Störungen im körpereigenen Abwehrsystem im Sinne einer immunologischen Dysregulation (Abb. 1). Gleichzeitig wurde für diese Erkrankungen ein Zusammenhang zwischen hoher psychosozialer Belastung und dem Krankheitsverlauf nachgewiesen.

Die Diskussion um das Post-Covid-Syndrom (PCS) motiviert zu einem frischen Blick auf dieses Phänomen. Insbesondere stellt sich die Frage, warum so viele Erkrankungen, bei denen die Immunantwort eine entscheidende Rolle spielt, sowohl auf Stress reagieren als auch mit der Entwicklung von Fatigue assoziiert sind. Außerdem interessiert, mit welchen Mitteln vor allem der Chronifizierung von Fatigue entgegengewirkt werden kann. Die Psychoneuroimmunologie ist eine relativ junge Forschungsrichtung, die hier interessante Ansätze zu bieten hat. Sie erforscht die engen Zusammenhänge zwischen psychosozialer Belastung und der Funktionsfähigkeit des Immunsystems. Therapeutische Ansätze, die eine bedarfsangemessene Stressantwort fördern, die also die Anpassung der Stressreaktionssysteme, einschließlich Immunsystems, an biopsychosoziale Herausforderungen optimal unterstützen, sind hier von besonderem Interesse.

Dieser Artikel bietet eine Übersicht zum Stand der wissenschaftlichen Erkenntnisse zu Fatigue, von ihrer Definition und Prävalenz über die Rolle der Immunantwort bis hin zum biopsychosozialen Kontext und zu psychoneuroimmunologischen Erklärungsmodellen und den Behandlungsmöglichkeiten von erhöhter Fatigue bei Verlust von Entzündungskontrolle. In diesem narrativen Review werden die bislang bekannten Zusammenhänge zwischen hoher biopsychosozialer Belastung, Fatigue und Immunantwort zusammenfassend dargestellt. Dabei wird herausgearbeitet, wie gerade chronische Entzündungsprozesse Erschöpfung produzieren, wie Stress diese Entzündungsprozesse fördert und wie therapeutisch auf diese Zusammenhänge eingewirkt werden kann.

## Was ist Fatigue und wie häufig tritt sie auf?

Als Fatigue wird im klinischen Alltag meist eine Erschöpfung bzw. Ermüdung bezeichnet, die sich unverhältnismäßig zum Ausmaß einer unmittelbar vorangegangenen körperlichen oder seelisch-geistigen Herausforderung verhält. Sie erhält dadurch Krankheitswert, dass die üblichen Maßnahmen zur Erholung kaum zur Regeneration beitragen und es zu einer deutlichen Einschränkung der Betroffenen in allen Lebensbereichen kommt. Zumindest bei einem Teil der Betroffenen erstreckt sich diese Erschöpfung nachweislich bis in den Bereich der Immunfunktion. Störungen der Immunantwort sind jedoch nicht regelhaft nachweisbar. Von der Fatigue ist die Fatigability – Erschöpfbarkeit – abzugrenzen. Fatigability beschreibt eine messbare Veränderung der Leistungsfähigkeit und kann je nach betroffenem Bereich durch klinische Untersuchungen objektiviert werden, z. B. über neuropsychologische Tests oder die Erfassung der Handkraft, die auch für die Diagnostik eines Chronic Fatigue Syndrom (CFS) genutzt wird [2]. Fatigability wird häufig im Zusammenhang mit chronisch destruktiven und immunologischen Störungen berichtet [3].

Fatigue kann also aktuell zwar mit einer breiten Palette von Selbstauskunftsinstrumenten gemessen werden [1], nicht jedoch mit eindeutigen klinischen Untersuchungen, Laboranalysen oder apparativen Verfahren. Es ist zurzeit noch schwierig, die somatischen und die psychosozialen Aspekte bei der Überschreitung der Belastungsgrenze einzuordnen und zu verstehen, welche Kräfte treibend sind und wie sie sich gegenseitig bei der Entwicklung und Aufrechterhaltung von Fatigue beeinflussen. Diese Einordnung ist bislang mit keinem der zur Verfügung stehenden Instrumente möglich. Die unbefriedigende diagnostische Situation führt nicht selten zu langen und frustranen Krankheitsbiografien, bei denen von Fatigue Betroffene nicht nur kaum Hilfe, sondern selten überhaupt Interesse an ihrem Zustand erfahren. Auch wenn anlässlich des jüngsten Deutschen Ärztetages einmal mehr konstatiert wurde, dass Zeit das höchste Gut in der medizinischen Versorgung ist, scheinen komplexe Symptombilder wie die Fatigue gerade diese jedoch häufig nicht zu bekommen [4]. Stattdessen werden Betroffene nicht selten mit stigmatisierenden Zuschreibungen konfrontiert, wie der Annahme, sie würden simulieren, müssten sich nur zusammenreißen, würden nicht genug für sich, ihre körperliche Fitness oder ihr Immunsystem usw. tun.

Damit wächst der Bedarf, Mechanismen der Erschöpfung besser zu verstehen und z. B. mit immunologischen Untersuchungen diagnostisch belegen zu können. Die Erfahrungen aus der COVID-19-Pandemie und die aktuellen Möglichkeiten multimodaler Datenerhebung bei von Fatigue betroffenen Patient*innen haben jetzt die Forschung auf diesem Gebiet intensiviert [5], sodass die Hoffnung besteht, dass in Zukunft Gruppen von Betroffenen identifiziert werden können, für die eine klare Pathogenese beschrieben werden kann und zielführende Therapieoptionen zur Verfügung stehen [6].

Über Fatigue als Symptomkomplex scheint in den letzten Jahren zunehmend berichtet zu werden, wobei hier sicher auch die Möglichkeiten der Onlinebefragungen und der vermehrte Einsatz von Fragebögen zur Erfassung von Fatigue im Rahmen von klinischen Studien eine Rolle spielen. Insbesondere im Rahmen der COVID-19-Pandemie wurde Fatigue häufig thematisiert. Mit großen Schwankungen zwischen den Studien zeigten im Rahmen der Pandemie sowohl Studien über die allgemeine Bevölkerung als auch speziell über Menschen, die COVID-19 durchmachten, Prävalenzen von bis zu 50 % [7]. Bei chronisch entzündlichen Erkrankungen wurden Prävalenzen von bis zu 70 % beschrieben [8]. Und ca. ein Viertel befragter Krebspatient*innen berichtete bis zu 10 Jahre nach ihrer Erkrankung und Behandlung von Fatigue [9].

## Welche Rolle spielt das Immunsystem bei Fatigue?

### Aufgabe eines gesunden Immunsystems: Homöostase

Um eine Einordnung der Rolle des Immunsystems bei Fatigue zu ermöglichen, ist es zunächst hilfreich, sich zu vergegenwärtigen, welche Aufgaben die körpereigene Abwehr hat. Ein gesunder Mensch ist fähig, im Schadensfall, wenn also z. B. eine Mikrobe eindringt, eine Verletzung entsteht oder ein Umweltschadstoff toxische Effekte entfaltet, eine Immunantwort aufzubauen, die den Schaden zunächst begrenzt, dann im Rahmen eines Lernprozesses die Schadensverursacher passgenau identifiziert und schließlich die Ursache sowohl nachhaltig beseitigt als auch den entstandenen Schaden repariert.

Um Schaden zügig zu begrenzen, werden zunächst angeborene Infektabwehrmechanismen aktiviert. Dabei bilden Haut und Schleimhäute eine erste mechanische und biochemische Barriere, die u. a. mithilfe von Säureschutzmantel und Mustererkennungsrezeptoren endogene Schadenssignale („danger-associated molecular patterns“ *–* DAMPs) unspezifisch erkennen und Beseitigungsmechanismen aktivieren. Alarmsignalkaskaden der angeborenen Immunantwort mobilisieren in der Folge innerhalb der ersten Stunden der Reaktion über Moleküle wie die Toll-like-Rezeptoren Zellen der angeborenen Immunität wie die Makrophagen, natürliche Killerzellen, Granulozyten oder Mastzellen [10]. Der Schaden wird von diesen Zellen lokal begrenzt über unspezifische Mechanismen wie eine H_2_O_2_-Freisetzung, Ödemgeneration, die Aktivierung von Blutplättchen und „neutrophil extracellular traps“ (englisch für neutrophile außerzelluläre Fallen, abgekürzt NETs) u. v. m. [10].

Diese initiale Reaktion verschafft zum Preis einiger Kollateralschäden die nötige Zeit für die bedarfsangepasste Aktivierung der adaptiven Immunantwort, für die die Antigen-präsentierenden Zellen und die Aktivierung von T‑Helferzellen (TH) zentral sind [10]. Je nach immunologischer Herausforderung übernehmen verschiedene Subpopulationen wie TH1, TH17, TH2 usw. sowie zytotoxische T‑Lymphozyten die punktgenaue Elimination der Schadensverursacher. Schließlich werden entzündungsbegrenzende T‑regulatorische Zellen und B‑ sowie T‑Gedächtniszellen auf den Weg gebracht, die den Entzündungsprozess nach erfolgreicher Schadenseliminierung terminieren und eine effizientere Reaktion in der Zukunft ermöglichen [10].

### Störung der immunologischen Homöostase bei Fatigue

**Abb. 2:**
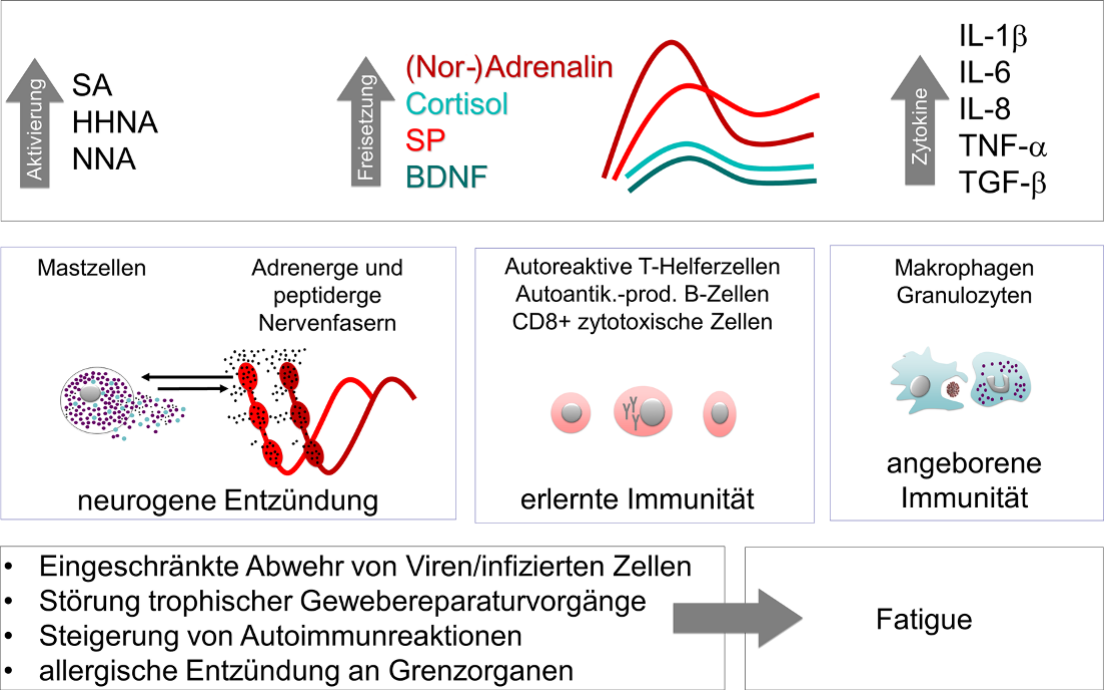
Hypothetisches Szenario der Entstehung von Fatigue durch neuroendokrinimmune Dysregulation bei länger anhaltendem Stress und wiederholter immunologischer Reizung. Die zentralnervöse sowie periphere Aktivierung der HHNA, SA und NNA erfolgt in einem Muster, das Viruselimination behindert, Gewebeschädigung verschärft und zu Fehlleistungen des Immunsystems führt. *BDNF* Brain Derived Neurotrophic Factor, *HHNA* Hypothalamus-Hypophysen-Nebennierenrinden-Achse, *IL* Interleukin, *NNA* Neurotrophin-Neuropeptid-Achse, *SA* sympathische Achse, *SP* Substanz P, *TGF* Tumor Growth Factor, *TNF* Tumornekrosefaktor. (Quelle: eigene Abbildung)

Bei Fatigue konnten bestimmte Veränderungen im Ablauf immunologischer Prozesse wiederholt nachgewiesen werden. Wie es nach einem Infekt, bei einer autoimmunen Erkrankung, einer Krebserkrankung oder einer psychischen Erkrankung zu anhaltenden oder auch zeitversetzt zu einer chronischen Immunfehlaktivierung kommen kann, ist jedoch bislang nur rudimentär geklärt [9]. Eine zentrale Hypothese verfolgt das Konzept, dass Fatigue durch eine Hyperaktivierung von angeborenen Immunmechanismen, man spricht auch von Aktivierung des proinflammatorischen Zytokinnetzwerkes, welches z. B. Interleukin (IL) 1β, IL‑6 und Tumornekrosefaktor-alpha (TNF-α) umfasst, verursacht wird ([11]; Abb. 2). Dabei kommt es zu überhandnehmenden Kollateralschäden an umliegenden gesunden Zellen.

Als Auslöser einer fortgesetzten Aktivierung der angeborenen Immunität gelten über längere Zeit wirksame Schaden verursachende biophysikalische, toxische, mikrobielle, onkologische und auch psychosoziale Reize. Sie können durch fortgesetzte entzündliche Aktivität zahlreiche mikroskopisch kleine Schäden verursachen, die insbesondere Organe mit hohem Zellaustausch oder mit geringer regenerativer Kapazität betreffen, also z. B. das Nervensystem, das Immunsystem, Endo- und Epithelien, Schleimhäute und Haarfollikel. Ein simpler klinischer Indikator für diesen Vorgang ist, dass 12 Wochen nach Infektion häufig vermehrter Haarausfall auftritt. Klinisch sichtbarer Haarausfall ist eine häufiges Spätsymptom vom systemisch wirksamen Entzündungsprozessen und wird durch proinflammatorische Zytokine initiiert [6]. Fatigue geht diesem sicht- und messbaren Symptom ankündigend voraus [12].

Mit der in der Routine verfügbaren apparativen und Labordiagnostik und ihrer relativ groben Auflösung sind bei Fatigue allerdings häufig keine eindeutigen Befunde zu erheben, die eine chronische Gewebeschädigung durch fortlaufende unterschwellige systemisch wirksame Entzündung belegen. Sie kann sich allerdings durch Surrogatparameter bemerkbar machen. So wird häufig eine Erhöhung des C‑reaktiven Proteins (CRP) nachgewiesen, wenn steigende Fatigue beobachtet wird, vor allem bei jener, die mit körperlichen Faktoren assoziiert ist. Eine andauernde Aktivierung der Immunantwort führt schließlich auch zu Fehlfunktionen der adaptiven Immunität, also z. B. zur messbaren Produktion von Autoantikörpern und Nachweis vermehrt auftretender autoreaktiver T‑Zellen [10]. Autoantikörper richten sich bei chronischen Entzündungsprozessen häufig gegen Rezeptoren, die bei der Stressreaktion aktiviert werden. Hierzu zählt z. B. der Beta-2-Adrenorezeptor [13]. Sie können für die autonome Dysregulation mitverantwortlich sein, die bei Fatigue häufig beobachtet wird. Doch ist Vorsicht vor Überinterpretation geboten: Betrachtet man z. B. Patient*innen mit Depression, dann korreliert Fatigue nicht notwendigerweise mit CRP und nur etwa ein Drittel der Patient*innen zeigt eine erhöhte Entzündungsaktivität in Form einer „low-grade inflammation“ [14]. Auch gelingt der Nachweis von Autoantikörpern und ihrer Assoziation mit Fatigue z. B. beim PCS nur bei einem Teil der Betroffenen [15]. Die beschriebenen immunologischen Mechanismen können also nicht regelhaft mit Fatigue assoziiert werden.

### Negative Energiebilanz bei chronischer Entzündung

Bei immunologischen Prozessen wird sehr viel Energie verbrannt, was zu metabolischer und oxidativer Überlastung führen kann. Entzündung verschiebt dabei den Energiemetabolismus in Richtung anaerober Hyperglykämie, was zu einer schnellen Erschöpfbarkeit der Energiebereitstellung beiträgt. Zu einer weiteren Beeinträchtigung der Energiebilanz kommt es, wenn Mitochondrien von metabolischer und oxidativer Überlastung betroffen sind [16].

Mitochondrien produzieren den größten Teil der Energie aller Körperzellen, einschließlich des Gehirns, der Muskultur und des Immunsystems. Eine Störung der Funktion dieser Zellkraftwerke oder auch schlicht ein Mangel an funktionsfähigen Mitochondrien beeinträchtig die Funktionalität und Resilienz betroffener Zellen und führt zu vorzeitiger Zellalterung. Beim CFS werden ein veränderter mitochondrialer Stoffwechsel und seine Rolle sowohl bei Virusinfektionen als auch bei Depression schon länger diskutiert [17]. Betroffene Zellen produzieren u. a. geringere Mengen des zentralen Energielieferanten Adenosintriphosphat (ATP), was bereits auf zellulärer Ebene zur Erschöpfung beiträgt [3]. Gleichzeitig kommt es bei gestörter Mitochondrienfunktion zum Auflaufen von schädigenden Stoffwechselabfallprodukten wie Sauerstoffradikalen, die zu oxidativen Stressschäden an Proteinen, Lipiden und DNA führen und damit zu einer weiteren Einschränkung der Zellfunktion und erhöhtem energieintensiven Reparatur- und Regenerationsbedarf führen. So kommt es bei Erkrankungen, die mit einer Schädigung der Mitochondrienfunktion einhergehen, zu vorzeitigen Alterungsprozessen und einer damit assoziierten Fatigue [18].

## Kann eine Dysregulation der Stressantwort die Funktion des Immunsystems beeinträchtigen und so zu Fatigue beitragen?

### Die Immunantwort ist in die Stressreaktion eingebunden

Die Stressreaktion und die Immunantwort sind über neuroendokrine Signale eng gekoppelt [10]. Neben einer angeborenen Immunantwort löst ein Schadensfall auch immer eine akute Stressreaktion aus. Diese wiederum ist immer an eine Mobilisierung der angeborenen Immunität gekoppelt. Dabei trägt die Ausschüttung von (Nor‑)Adrenalin und Cortisol zur Mobilisierung von Zellen der angeborenen Immunantwort bei.

Weniger bekannt ist, dass gleichzeitig von sensorischen und autonomen Nervenfasern Neuropeptide wie die Substanz P (SP; Abb. 2) und andere Neuropeptide wie das vasoaktive intestinale Peptid (VIP) im Gewebe freigesetzt werden. Diese neuronalen Stressmediatoren aktivieren vor allem in Organen an der Grenze zwischen Körper und Umwelt (Haut, Gastrointestinaltrakt, Atemwege) die vor Ort ansässigen Zellen des Immunsystems, wie z. B. die Mastzellen. Sie rekrutieren zudem Funktionszellen in den Organen für die Immunantwort, z. B. blutgefäßauskleidende Endothelzellen, bindegewebige Fibroblasten oder die barrierebildenden Keratinozyten.

Über die Ausschüttung neuroendokriner Mediatoren wie Cortisol, (Nor‑)Adrenalin, SP und bestimmter Neurotrophine begünstigt Stress das Durchdringen der Haut- und Schleimhautbarriere durch Schadstoffe, reduziert die Mikrobiomvielfalt und aktiviert weitere Zellen der angeborenen Immunantwort wie die Mastzellen, Makrophagen und Granulozyten [10]. Auch die Blut-Hirn-Schranke wird in ihrer Barrierefunktion beeinträchtigt, sodass es bei steigender peripherer Entzündung auch im Gehirn zu einer Immunaktivierung kommt, für die die Mikroglia eine zentrale Rolle spielt [19]. Gleichzeitig fungieren bestimmte Areale im Gehirn als Immunsensoren und werden durch proinflammatorische Zytokine aus der Peripherie aktiviert, allen voran die Insula und das Striatum. Diese Aktivierung modifiziert die Funktion der Hypothalamus-Hypophysen-Nebennierenrinden-Achse (HHNA) und der sympathischen Achse im Gehirn und beeinflusst auch die Dopaminausschüttung [20]. Es kommt zum Anstieg des Transkriptionsfaktors NF-κβ und von Zytokinen wie IL‑1, IL‑6 oder TNF‑α, peripher und zentral, also den proinflammatorischen Zytokinen, die bei Fatigue erhöht nachgewiesen werden können.

Im Gehirn führt der Anstieg dieser Zytokine u. a. zum Abfall des Neurotrophins *Brain Derived Neurotrophic Factor* (BDNF; [21]) und ruft ein im englischen Sprachraum als „sickness behavior“ bezeichnetes, depressionsähnliches Verhaltensmuster auf [22]. Im deutschen Sprachraum wird dieser Begriff im wissenschaftlichen Kontext häufig ohne Übersetzung verwendet, da Begriffe wie „unspezifische Krankheitssymptome“ oder „unspezifische Begleiterscheinungen von Erkrankungen“ das Symptombild nicht ganz erfassen, welches aus Schwäche, allgemeinem Unwohlsein, Schmerz, Schlaf- und Appetitstörungen, Verlust von Motivation und Konzentrationsfähigkeit sowie affektbezogenen Symptomen wie Traurigkeit und Ängstlichkeit besteht und das sich in anlassbezogener reduzierter körperlicher und geistiger Aktivität sowie Rückzugs- und Schonverhalten äußert.

Schließlich kann die veränderte Ausschüttung von Stressmediatoren wie Cortisol, (Nor‑)Adrenalin, SP oder BDNF die zeitgerechte Terminierung einer angeborenen Immunantwort blockieren. Wenn durch chronischen Stress das Morgencortisol reduziert ausgeschüttet wird, sind häufig auch die adrenerge und die peptiderge Schiene hyperreaktiv und eine Gegensteuerung durch Neurotrophine und cholinerge Signale bleibt aus (Abb. 2). Diese neuroendokrinimmunologische Konstellation blockiert die Infektabwehr genauso wie die Abwehr von Tumorzellwachstum, ein stetig ablaufender immunologischer Prozess, der, aus dem englischen Sprachgebrauch übernommen, auch als Tumor-Immune-Surveillance bezeichnet wird. Zusätzlich wird eine Hyperaktivität bestimmter adaptiver Immunprozesse, wie z. B. der Antikörperproduktion, begünstigt, was zur Entwicklung von Autoimmunreaktionen und Allergien beiträgt [23].

### Fatigue und Immundysregulation werden durch psychosoziale Belastung synchron beeinflusst

Interessanterweise verschärfen sich sowohl Fatigue als auch die oben beschriebene neuroendokrinimmunologische Dysregulation bei Patient*innen mit Infektionen, Krebs oder chronisch metaboentzündlichen Erkrankungen (z. B. Diabetes mellitus, autoimmunen und allergischen Erkrankungen, multipler Sklerose), wenn sie Kindheitstraumata oder Depression in der Vorgeschichte aufweisen oder unter Einsamkeit leiden [24, 25]. Psychosoziale Belastungen in der Vorgeschichte und damit chronische Stressoren unterschiedlicher Qualität begünstigen also synchron sowohl immunologische Entgleisung als auch die Entwicklung von Fatigue, was auf gemeinsame, transdiagnostische Mechanismen hinweist.

Umgekehrt zeigt der Einfluss von Entzündung auf Fatigue und psychosoziale Faktoren, das es sich hier nicht um eine einseitige Beziehung handelt, denn experimentell kann Fatigue mithilfe des bakteriellen Toxins Lipopolysacharid (LPS) ausgelöst werden, einer Substanz, die zuverlässig in gesunden Individuen proinflammatorische Zytokine mobilisiert und gleichzeitig „sickness behaviour“ auslöst [26].

Erste Hinweise auf die klinische Relevanz von Entzündung für die Entwicklung von Fatigue und psychosozialer Belastung ergaben sich aus der immuntherapeutischen Behandlung mit Interferon-alpha (IFN-α) bei Patient*innen mit chronischen Infektionskrankheiten oder schwarzem Hautkrebs [27]. Ein substanzieller Anteil dieser Patient*innen wies mit steigenden Inflammationsparametern, allen voran IL‑6 und TNF‑α, auch vermehrt Fatigue und depressive Symptomatik auf. Es brauchte nach Einführung dieser Medikamente jedoch einige Jahre, bis die beobachtete steigende psychopathologische Symptomatik zu Konsequenzen in der Anwendungsbreite der Behandlung führte. Zum Teil ist dies erklärbar dadurch, dass zwar eine klare Korrelation zwischen steigenden Zytokinen und Fatigue beobachtet werden konnte. Patient*innengruppen mit Fatigue zeigten im Vergleich zu solchen ohne jedoch nicht notwendigerweise auch erhöhte Zytokinwerte [28]. Es besteht also keine vollständige Reziprozität zwischen berichteter Fatigue und immunologischen Befunden.

Bei Krebspatient*innen wurden in den vergangenen Jahren die neuroendokrinimmunologischen Konsequenzen von gleichzeitiger körperlicher und psychosozialer Belastung mit am besten untersucht [29]. Dabei fand sich bei von Fatigue betroffenen Patient*innen als Hinweis auf eine Störung der Stressreaktivität eine Veränderung in der Funktion der HHNA, mit einer morgens abgeschwächten und abends erhöhten zirkadianen Ausschüttung von Cortisol bis zu 5 Jahren nach Diagnose [30]. Gleichzeitig fanden sich erhöhte Interferone sowie IL-1β, IL‑6 und TNF‑α als Indikatoren einer überaktiven angeborenen Immunität [31]. Als zusätzliche Zeichen einer fehlgeleiteten erlernten Immunität fanden sich aktivere antikörperproduzierende B‑Zellen, erhöhte CD8+-zytotoxische T‑Zellen und eine abgeschwächte T‑regulatorische Funktion. Eine pathogenetische Herleitung von Fatigue bei Krebspatient*innen führt den Anstieg von Erschöpfung daher entlang der oben skizzierten psychoneuroimmunologischen Erkenntnisse darauf zurück, dass eine gestörte Funktion der Stressreaktionssysteme Hand in Hand mit einer fortschreitenden Zerstörung von Gewebe bei Tumorprogression und bei zytotoxischer Behandlung auftritt. Dabei werden zum einen verstärkt proinflammatorische Zytokine freigesetzt und zum anderen entwickelt sich eine dysfunktionale erlernte Immunität. Gemeinsam fördern diese Prozesse wie oben beschrieben „sickness behaviour“ und vorzeitige Zellalterung.

Diese Vorgänge entsprechen Prozessen, die auch bei chronischen Infektionen und Stress beschrieben wurden. Interessant ist in diesem Zusammenhang, dass im Rahmen der Untersuchungen zum PCS, bei dem Fatigue besonders häufig beobachtet wird, langfristig niedrige Morgencortisolwerte die höchste prädiktive Kapazität für die Entwicklung des Syndroms zeigen [32] und korrespondierende immunologische Veränderungen beobachtet werden [33]. Vor dem Hintergrund der Interaktion von Stress und Immunantwort scheint Fatigue unter dem Einfluss von psychischer Belastung also durch eine initial nicht erfolgreiche angeborene Immunantwort begünstigt zu werden, wodurch die erlernte Immunantwort fehlaktiviert werden kann und es insgesamt zu langfristig erhöhten Inflammationsparametern, Erschöpfung von Mechanismen der Energiebereitstellung und erhöhtem zellulären Regenerationsbedarf kommt.

## Möglichkeiten, den Stress-Immundysregulation-Fatigue-Kreislauf zu durchbrechen

Entsprechend den weitreichend wirksamen biopsychosozialen Interaktionen bei Fatigue liegt es nahe, dass auch eine breite Palette von therapeutischen Ansätzen synchron auf erhöhte inflammatorische Aktivität und Fatigue einwirken kann. Die Studien, die eine Verbesserung von Fatigue und gleichzeitig Indikatoren für eine Rückführung einer maladaptiven Immunantwort in eine adaptive zeigen, reichen von Maßnahmen zur Optimierung eines gesunden Lebensstils über Entspannungsverfahren bis hin zu Psychotherapie und Psychopharmakotherapie. Es ist für eine zielführende Einschätzung des Therapiebedarfs wichtig zu beachten, dass hohe Inflammationswerte Indikatoren für ein reduziertes Therapieansprechen von Patient*innen mit Fatigue darstellen. Dies spricht für eine höhere Behandlungsbedürftigkeit von Patient*innen mit einer höheren Krankheitsschwere.

### Immunmodulation.

Aktuell wird der Einsatz von Biologica, meist Antikörper-basierte Medikamente, die individuelle Zytokine neutralisieren, auch für Fatigue diskutiert [34]. TNF-α- und Interferon-α-neutralisierende Behandlungen führen in der Tat laut zahlreichen Berichten zur Verbesserung von Schlafmustern und Fatigue. Die immunologischen Veränderungen bei Fatigue sind jedoch sehr umfangreich und komplex und nicht auf die Fehlregulation eines einzelnen Zytokins zurückzuführen, sodass mit überwiegend unerwünschten Effekten dieser Medikamente bei multifaktoriell ausgelöster Fatigue zu rechnen ist, ähnlich wie bei entsprechenden Behandlungsversuchen bei der Sepsis.

### Bewegung.

Galt in den 1980er-Jahren Bewegung noch für gesundheitsschädlich bei Erschöpfung auslösenden Krebserkrankungen, ist der therapeutische Nutzen von Bewegungstherapie inzwischen sowohl für die Krebs-assoziierte Fatigue als auch Tumor-Immune-Surveillance sehr gut belegt [35]. Auch bei Autoimmunerkrankungen und depressiver Symptomatik verbessert Bewegung Fatigue und immunologische Imbalancen [36]. Bei CFS wird im Rahmen der postexertionellen Malaise (PEM) Bewegung jedoch häufig als Auslöser von Fatigue beschrieben. So kann schon ein diagnostisches kardiopulmonales Training eine deutliche Fatigue-Verschärfung bewirken [37].

### Ernährung.

Was wir essen, hat einen nicht unerheblichen Einfluss auf chronische Entzündungsprozesse einerseits und psychosoziale Gesundheit andererseits. Eine entzündungshemmende Ernährungsweise kann sich günstig auf Fatigue auswirken. Sie enthält Omega-3-reiche Pflanzenöle, viel pflanzliche Eiweiße und überwiegend komplexe Kohlenhydrate, z. B. aus Vollkorn [38].

### Schlaf.

Ob Schlafstörungen und nicht erholsamer Schlaf Auslöser oder Folgen von Fatigue sind, ist umstritten. Nicht erholsamer Schlaf ist jedoch klar mit einer breiten Palette neuroendokrinimmunologischer Störungen assoziiert [39]. Maßnahmen zur Verbesserung der Erholung durch Schlaf erscheinen also angezeigt und können umfassend der AWMF (Arbeitsgemeinschaft wissenschaftlicher Medizinischer Fachgesellschaften) S3-Leitlinie „Nicht erholsamer Schlaf/Schlafstörungen – Insomnie bei Erwachsenen“ [40] entnommen werden. Eine Verbesserung von Schlaf geht mit einer Verbesserung von Fatigue häufig Hand in Hand [41].

### Psychische Gesundheit.

Fatigue kann als Schutzmechanismus des Körpers verstanden werden, der bei Überlastung zur Schonung und Fokussierung auf Schadensreparatur führt. Die niedrige Lebensqualität bei Fatigue, die erst kürzlich wieder für das PCS belegt wurde [42], weist jedoch deutlich darauf hin, dass auch die psychischen Aspekte von Fatigue zu behandeln sind. Die Studienlage zu psychotherapeutischen Verfahren ist entsprechend dem erforderlichen Goldstandard für Evidenzbasierung am besten für kurzzeittherapeutische Konzepte, allen voran manualisierte kognitiv-behaviorale Therapien (CBT). Für die Krebs-assoziierte Fatigue ist der therapeutische Nutzen von kognitiver Verhaltenstherapie und achtsamkeitssteigernden Therapieansätzen am besten untersucht [43]. Für das PCS liegen erste Arbeiten für integrierte stationäre Kurzzeittherapie und Rehabilitation [44, 45] vor. Sie können zudem für die Behandlung von Fatigue-assoziierten Symptomen wie der Insomnie erfolgreich eingesetzt werden [46]. Für diese psychotherapeutischen Behandlungsansätze ist zudem eine Senkung proinflammatorischer Zytokine sehr gut belegt. Sie können nachweislich immunologische Imbalancen reduzieren und die Immunantwort flexibilisieren. Metaanalysen zeigen, dass dabei z. B. zirkulierende Zytokine wie das IL‑6 und das TNF‑α reduziert werden und eine adäquate Antikörperantwort gefördert wird [47].

Im Kontext von Fatigue und depressiver Symptomatik, die durch Interferontherapie hervorgerufen wird, konnte gezeigt werden, dass die Symptome gut auf die Behandlung mit Antidepressiva ansprechen. Im Tiermodell erwies sich dieses Vorgehen auch wirksam bei entzündungsassoziierter Fatigue, ausgelöst z. B. durch das bakterielle Toxin Lipopolysacharid (LPS). Hier sind entsprechende Medikamente auch wirksame Dämpfer der Mikrogliaaktivierung [48]. Insbesondere Buproprion, ein Noradrenalin-Dopamin-Wiederaufnahmehemmer, Paroxetin, ein Serotonin-Wiederaufnahmehemmer, und das tetrazyklische Antidepressivum Mirtazapin können hier, wie auch bei Krebs-assoziierter Fatigue, wirksam zur Senkung zirkulierender Zytokine und der Fatigue-Symptomatik eingesetzt werden [27]. Dabei produzieren interessanterweise ex vivo untersuchte Zellen des Immunsystems unter Behandlung mit diesen Antidepressiva vermehrt Zytokine. Insgesamt scheint also einerseits systemische Hyperinflammation gesenkt zu werden, Zellen des Immunsystems werden ggf. jedoch gleichzeitig nicht pauschal immunsupprimiert, sondern in die Lage versetzt, wieder adäquat auf Reize zu reagieren. Offen bleibt vor dem Hintergrund der aktuellen Datenlage, ob die erfolgreiche Adressierung von Fatigue mit der Gabe von Antidepressiva primär auf der Ebene des Erlebens und Leidens an der Erschöpfung ansetzt oder auf der Ebene der Zytokine.

## Fazit

Fatigue ist ein biopsychosozial zu betrachtendes pandiagnostisches Symptom bei Erkrankungen mit immunologischer Dysregulation, weist aber nicht notwendigerweise auf eine Dysregulation hin. Insbesondere Fatigue mit nachweislichen immunologischen Veränderungen kann sowohl bei Infektions-, onkologischen und internistischen Erkrankungen als auch bei psychischen Erkrankungen, insbesondere bei der schweren Depression, beobachtet werden. Dabei ist Fatigue vor allem bei Erkrankungen mit langen Verläufen beschrieben worden, nicht selten auch als Frühzeichen einer zugrunde liegenden Erkrankung. Zur Unterscheidung verschiedener mit Fatigue assoziierter Krankheitsverläufe ist eine umfassende fachgerechte Diagnostik angezeigt. Damit können von Fatigue belastete Patient*innen frühzeitig identifiziert werden und zügig geeignete Behandlungen für alle Symptome bekommen, die zur Einschränkung der Lebensqualität der Patient*innen beitragen.
